# Antioxidant and Antimicrobial Properties of Rosemary (*Rosmarinus officinalis,* L.): A Review

**DOI:** 10.3390/medicines5030098

**Published:** 2018-09-04

**Authors:** Gema Nieto, Gaspar Ros, Julián Castillo

**Affiliations:** 1 Department of Food Technology and Human Nutrition, Veterinary Faculty, University of Murcia, Espinardo, 30071 Murcia, Spain; gnieto@um.es (G.N.); gros@um.es (G.R.); 2 Research and Development Department of Nutrafur-Frutarom Group, Camino Viejo de Pliego s/n, Alcantarilla, 80320 Murcia, Spain

**Keywords:** rosemary, phenolic compounds, antioxidant, antimicrobial

## Abstract

Nowadays, there is an interest in the consumption of food without synthetic additives and rather with the use of natural preservatives. In this regard, natural extracts of the *Lamiaceae* family, such as rosemary, have been studied because of its bioactive properties. Several studies have reported that rosemary extracts show biological bioactivities such as hepatoprotective, antifungal, insecticide, antioxidant and antibacterial. It is well known that the biological properties in rosemary are mainly due to phenolic compounds. However, it is essential to take into account that these biological properties depend on different aspects. Their use in foods is limited because of their odour, colour and taste. For that reason, commercial methods have been developed for the preparation of odourless and colourless antioxidant compounds from rosemary. Owing to the new applications of natural extracts in preservatives, this review gives a view on the use of natural extract from rosemary in foods and its effect on preservative activities. Specifically, the relationship between the structure and activity (antimicrobial and antioxidant) of the active components in rosemary are being reviewed.

## 1. Introduction

Because consumers are concerned about the negative effect of synthetic chemicals in food, there is a need to find “clean label products”. Therefore, there is a growing interest in using natural extracts as alternatives for synthetic additives because of (a) their synergy with other preservation methods (b) they are considered safe, and (c) their specific properties as antioxidant, antidiabetic, antimutagenic, antitoxigenic and antibacterial [1].

In general, herbs and plants are rich in compounds with antioxidant properties, such as vitamins (E and C), glutathione, enzymes and phenolic compounds [2]. Several spice extracts have shown their properties to prevent the autoxidation of unsaturated triacylglycerols [3]. Specifically, the natural extract from the *Lamiaceae* family (thyme, sage and rosemary) has been reported in several studies for its antioxidative activity [1,4].

*Rosmarinus officinalis*, L. originating from the Mediterranean region is an aromatic plant from the *Lamiaceae* family. The province of Murcia (Southeast Spain) is one of the major processors and importers of rosemary. In the United States and Europe, rosemary is a unique spice commercially available for use as an antioxidant [5]. Rosemary extracts have been used in the treatment of diseases, due to its hepatoprotective potential [6], therapeutic potential for Alzheimer’s disease [7] and its antiangiogenic effect [8]. On the other hand, they have been used in food preservation, because they prevent oxidation and microbial contamination [9,10,11,12,13]. Therefore, rosemary extract could be useful for replacing or even decreasing synthetic antioxidants in foods. As preservatives, rosemary extracts offer several technological advantages and benefits to consumers.

EFSA (European Food Safety Authority) has reviewed the safety of rosemary extracts [14]. It concluded that there are high-intake estimates ranging from 0.09 (the elderly) to 0.81 (children) mg/kg per day of carnosol and carnosic acid. Nowadays, in the European Union, rosemary extracts are added to food and beverages at levels of up to 400 mg/kg (as the sum of carnosic acid and carnosol).

## 2. Preparation of Antioxidants from Rosemary

When a new rosemary extract is tested, the most important aspect to take into account is the method of extraction and the sort of solvent used, as this will affect the antioxidant properties.

Various extraction methods for the selective extraction of rosemary leaves have been identified in the scientific literature. This includes both solvent extraction using vegetable oil or animal fat, mechanical pressing methods, water at alkaline pH and organic solvents (e.g. hexane, ethyl ether, chloroform, ethanol, methanol, dioxane and ethylene dichloride).

Previous extraction processes are obsolete and unusual in the industry. Chang et al. [15] and Bracco et al. [16] demonstrated the possibility of separating an active fraction of the rosemary extract by molecular distillation. The process realized by Chang et al. [15] for the extraction of antioxidant involves the extraction with ethyl ether under refluxing conditions. The crude material is washed with water and the solvent is removed. The crude material is dissolved in methanol and activated with carbon at 60 °C for 15 min. Another process reported by Bracco [16], involves reducing the particle size to 600 µL and suspending in peanut oil. The finely antioxidant components have a molecular weight range lower than the triglyceride components of the oil used (peanut oil) and can therefore be physically separated by molecular distillation on either a fall-film or centrifugal system.

Nowadays, rosemary extracts are usually prepared from dried rosemary leaves. In all the new methods, the extraction process is often accompanied by a step involving partial deodorisation and/or extract decolourisation. Considering the methods used, in general, the yield of rosemary extract indicated by various authors varies between 2% and 26% based on the raw material used.

## 3. Composition of Rosemary Extract and Essential Oil

The polyphenolic profile of rosemary has been widely described in the scientific literature [17,18,19,20]. The polyphenolic profile of these plants is characterized by the presence of carnosic acid, carnosol, rosmarinic acid and hesperidin, as major components [21].

Among the most effective antioxidant constituents of rosemary, the cyclic diterpene diphenols, carnosolic acid and carnosol have been identified. In addition, its extract contains carnosic acid, epirosmanol, rosmanol, methylcarnosate and isorosmanol [21,22,23].

Rosemary oils are obtained by steam distillation of twigs and fresh leaves. Sienkiewicz et al. [24] reported that rosemary essential oil contains mainly 1,8-cineole (46.4%), camphor (11.4%) and α-pinene (11.0%). The composition of the rosemary essential oil used by Jiang et al. [25], was composed mainly by 1,8-cineole (26.54%) and α-pinene (20.14%). Bendeddouche et al. [26], observed that the main constituents of the tested essential oil were camphor (37.6%), 1,8-cineole (10.0%), p-cymene-7-ol (7.8%) and borneol (5.4%).

*Rosmarinus officinalis*, L. is a rich source of phenolic compounds and their properties are derived from its extracts [27] and essential oils [28]. Both are used for the treatment of illnesses and in the food preservation.

In addition to the volatile constituents, extracts of rosemary also contain several antioxidant components, which belong mainly to the classes of phenolic acids, flavonoids, and diterpenoids. Del Baño et al. [2] studied the composition of seven flavonoids in rosemary leaves, flower, roots and steam. They studied the presence of 7-*O*-glucoside, hispidulin, diosmin, hesperidin, 3′-o-β-d-glucuronide, genkwanin and isoscutellarein 7-*O*-glucoside. These authors concluded significant presence of diosmin and hesperidin in the vascular system. Of the other identified compounds found in extracts of rosemary, rosmarinic acid and hydroxyhydrocaffeic acid, also exhibit some complementary antioxidant activity.

The extract of rosemary also contains other caffeic acid derivates. These compounds react with present metal ions, so chelates are formed; they consequently react with peroxide radicals and in that way, stabilise these free radicals.

Rosemary oil is used as a food seasoning [29], due to its chemical compound constituents responsible for the antibacterial, antifungal and antioxidant properties. Traditionally, rosemary oil has been shown to possess a number of applications in managing or curing many diseases such as inflammatory diseases [30] and diabetes mellitus [31].

On the other hand, the bioactivities of rosemary extracts include properties such as anti-inflammatory [32], antidiabetic [33], hepatoprotective [34] and antimicrobial activity [35]. These bioactivities are related to the phenolic compound constituents (mainly caffeic acid, rosmarinic acid and carnosic acid).

## 4. Mechanism of Antioxidant Action

The mechanism of action of these compounds has been widely covered in several publications. For example, Höulihan et al. [36] and Wu et al. [37] determined that the antioxidant properties of rosemary are attributed to its richness in isoprenoid quinones, which act as chain terminators of free radicals, and as chelators of reactive oxygen species (ROS). In addition, Gordon [38] indicated that the phenolic compounds existing in the commercial extracts of rosemary act as primary antioxidants when reacting with the lipid and hydroxyl radicals to turn them into stable products. Subsequently, Fang and Wada [39] pointed out that these compounds could act as metal ion chelators (Fe^+2^ fundamentally), therefore reducing the formation ratio of the reactive species derived from oxygen.

According to Löliger [40], carnosic acid and carnosol act as potent scavengers of peroxyl radicals. This fact explains the conclusions obtained by Chen et al. [41], who confirmed that the effect of both compounds on peroxidation of membrane lipids is higher than the effect reported by artificial antioxidants such as BHA, BHT and propyl gallate [42].

The antioxidative activity of rosemary extracts has been evaluated using different solvents. In this regard, Inatani et al. [43] reported that rosmanol, showed an antioxidant capacity four times higher than BRT and BRA (synthetic antioxidants) in both linoleic acid and lard. In addition, this study reported the antioxidant activity of carnosol and rosmanol by TBA and ferric thiocyanate methods. They reported the correlation between activity and chemical structure as an antioxidant. Aruoma et al. [44] studied the antioxidant and pro-oxidant properties of rosemary. The main constituents with antioxidant properties are carnosic acid and carnosol that are responsible for 90% of the properties. Both are inhibitors of lipid peroxidation in liposomal and microsomal systems, they are good scavengers of CCl_3_O_2_ (peroxyl radicals), reduce cytochrome c and scavenge hydroxyl radicals. Specifically, carnosic acid scavenges H_2_O_2_, but could also act as a substrate for the peroxidase system.

**Figure 1:**
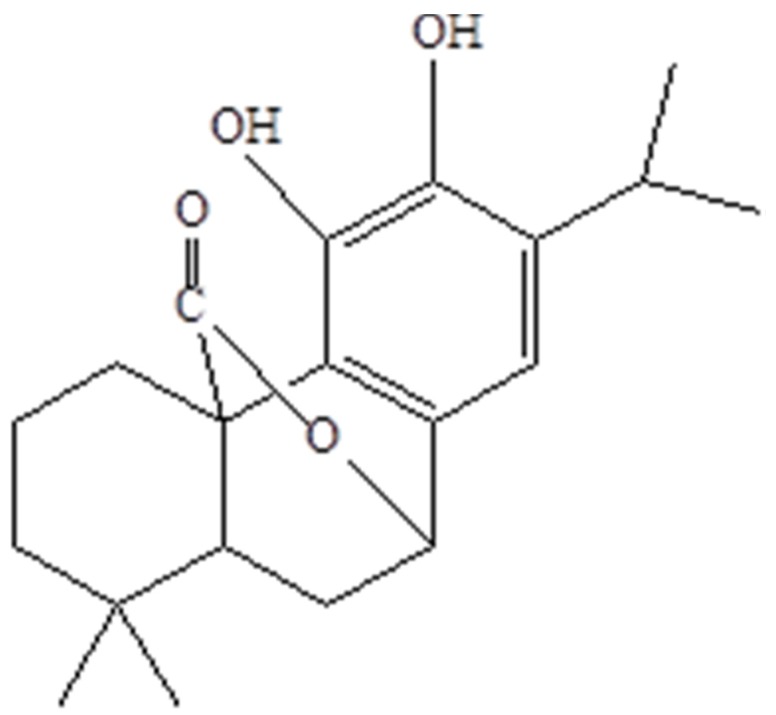
The chemical structure of carnosol.

The antioxidant properties depend on fruiting stages: the increase in concentration of polyphenols, which include carnosol, rosmarinic acid and hesperidin, during the fruiting stage, is directly related to the improvement of the extract antioxidant capacity. This statement is supported by scientific papers previously published by Cui et al. [45] and Kontogianni et al. [20], who consider lactone carnosol (Figure 1) as the main property responsible for this activity. Likewise, rosmarinic acid and hesperidin have been cited in literature as important free radical scavengers [46,47].

One of the most significant aspects of the antioxidant activity of rosemary is the relationship between diterpenes and radical-scavenging activity. In this regard, the study by Munné-Bosch and Alegre [48] describes the antioxidant capacity of diterpenes in rosemary. The most important elements in the rosemary structure are the aromatic ring (C_11_–C_12_) in the catechol group together with the conjugation of the three basic rings.

The catechol group is responsible for scavenging the radical electron formed as result of oxidation. The skeleton formed by the three rings allows the delocalization of the charge. The presence of the carboxylic group (in the case of carnosic acid) increases this conjugation, especially in aqueous systems. However, in slightly polar media, such as fats, it is the lactone structure that seems to confer greater stability.

**Figure 2:**
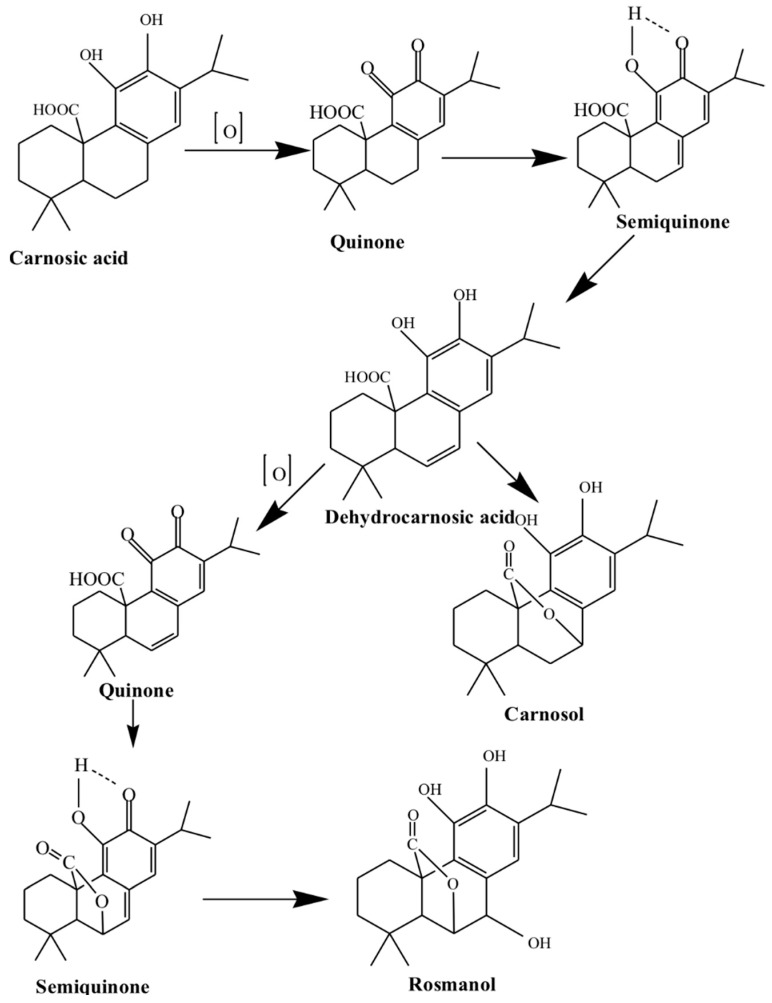
The first proposed oxidation pathway of carnosic acid. Reproduced from [49]. Copyright 1965, American Chemical Society, Washington, DC, USA.

Moreover, activation of redox-dependent signalling pathways such as Nrf2-dependent transcriptional regulation is known to take part in the antioxidant response of rosemary. The main metabolites carnosol and carnesolic acid have been reported to mediate antioxidant activities by differential mechanisms. Carnosic acid, carnosol, rosmanol and epirosmanol are the major phenolic diterpenes responsible for the antioxidant properties of rosemary (the first proposed oxidation pathway of carnosic acid was reported by Wenkert et al. [49] as shown in Figure 2). In the same way, Wijeratne and Cuppett [50] suggest that the antioxidant activities of carnosic acid are due to their ability to increase or maintain superoxide dismutase and glutathione peroxidase activities. These authors reported that carnosic acid and carnosol inhibited lipid peroxidation by 88–100% and 38–89%, respectively, under oxidative stress conditions.

Generally, the antioxidant effectiveness of natural extracts is higher than synthetic antioxidants, independent of the medium, which is different in water or oil. Table 1 shows the relative antioxidant effectiveness of spices and herbs.

For reasons explained in past years, the use of new rosemary extracts, mainly refined extracts with antioxidant properties, is an interesting strategy as a food preservative, especially those with animal and/or vegetable fats [16].

There are different applications of rosemary in foods. For example, it has been added to animal products and oils. Different studies have demonstrated the potent activity of rosemary by reducing the colour loss of carotenoids and delaying lipid oxidation in oils [55] and meat products [56,57,58].

Several studies have shown rosemary properties to achieve good sensory results and the reduction of lipid oxidation after addition into foods: Stoick et al. [59] used 500–1000 ppm of rosemary extracts in beef; Shahidi et al. [60] used rosemary at 200 and 1000 ppm in different foods; Huisman et al. [61] used a concentration of 0.05% in pork; Sánchez-Escalante et al. [62] used a combination of 500 ppm vitamin C and 1000 ppm of rosemary in beef burgers; Formanek et al. [63] used 0.25% of rosemary in beef burgers. In general, all these authors showed that rosemary inhibited the formation of hydroperoxides [64].

On the other hand, rosemary extract could be applied through animal diet. In this regard, delayed lipid oxidation was reported in broilers with the administration of sage and rosemary [65]. This agreed with the results of Moñino et al. [66], indicating that antioxidant stability improved with 10% of rosemary leaves into the ewes feed.

The antioxidant activity of rosemary through animal diet has been reported in additional studies by: Lopez Bote et al. [65] with 500 mg kg^−1^ in broiler diets; Descalzo et al. [67] in feed of cattle; Petron et al. [68] in lamb, in pork meat [69,70], turkey meat products [71,72], chicken meat [65], hen’s meat [73,74], cooked sausages [75]. Generally, the addition of rosemary extract into the meat products or through animal feed improved the meat lipid stability. On the contrary, O’Grady et al. [76] and Galobart et al. [77] concluded that feeding animals with rosemary did not improve the lipid stability of meat or eggs.

As previously reported, the antioxidant effect of rosemary is due to the polyphenols present in the leaves (mainly rosmarinic acid, carnosol and carnosic acid), which accumulate in the fatty membranes of cells where the antioxidant effect is required [72].

Other studies reported the effectiveness of rosemary oil to control oxidation in frankfurters [78] and to protect protein oxidation of meat patties [79]. This behaviour is very important to control colour changes of meat products mainly caused when the haem pigments are oxidized. In this regard, lipid oxidation and their consequences are reduced through the addition of rosemary.

In addition, rosemary has been added as an antioxidant in oils: Tohma et al. [80] studied the effect of the rosemary plant, its alcoholic extracts and essential oil on the oxidative stability of hazelnut oil during deep frying. This study showed that rosemary plant, or its extracts, could be used to extend the usage life of hazelnut oil for frying. The rosemary additives considerably inhibited the formation of oxidation products. The total phenolic compounds and profile of the enriched oils might cause increased frying stability. In addition, Reblova et al. [3] and Taha et al. [81] reported that rosemary extracts improved the sensory characteristic of fries, increased the oxidative stability of the oil, inhibited the decomposition of polyunsaturated triacylglycerols and the formation of polar substances in rape-seed oil. Urbancic et al. [82] reported that rosemary extract reduced acrylamide formation during potato frying. This effect was due to the transfer of phenolic compounds from rosemary into the oil.

## 5. Mechanism of Antimicrobial Action and Food Applications

The antibacterial activity of rosemary has been determined in various assay types based on either MIC or MBC. In this regard, Sienkiewicz et al. [24], demonstrated the antibacterial activities of basil (*Ocimum basilicum*, L.) and rosemary (*Rosmarinus officinalis*, L.) These authors reported the inhibition of microbial growth by both essential oils, presented as MIC values. Antibiotic susceptibility was carried out using disc diffusion. The results showed that both essential oils tested are active against all the clinical strains from *Escherichia coli*. Mihajilov-Kristev et al. [83], showed that essential oils containing mainly carvacrol (67.0%) and γ-terpinene (15.3%) were effective against Gram-negative strains, including *Escherichia coli*, with MIC values from 0.025 µL/mL to 0.78 µL/mL according to the broth microdilution method. Probuseenivasan et al. [84], confirmed that rosemary essential oil strongly inhibits *E. coli* ATCC 25922. The minimal inhibitory concentration for rosemary oil against *E. coli* was >6.4 mg/L.

Other studies have shown the antibacterial activity of rosemary oil against *E. coli*, *Bacillus cereus*, *Staphylococcus aureus* [85], *Staphylococcus aureus*, *Clostridium perfringens*, *Aeromonas hydrophila*, *Bacillus cereus* and *Salmonella choleraesuis*. This essential oil was incorporated into meat reporting antibacterial activity against *Brochothrix thermosphacta and Enterobacteriaceae* [86].

The inhibitory effect of rosemary is the result of the action of rosmarinic acid, rosmaridiphenol, carnosol, epirosmanol, carnosic acid, rosmanol and isorosmanol. They interact with the cell membrane, causing changes in genetic material and nutrients, altering the transport of electrons, leakage of cellular components and production changes in fatty acid. In addition, it also produced an interaction with the membrane of proteins that produced the loss of membrane functionality and its structure [87].

Vegara et al. [88] reported that the effectiveness of carnosic acid against pathogenic bacteria is superior to that of any other major extract component, including rosmarinic acid. In contrast, several scientific publications disagree about the possible relationships that may exist between the composition of the polyphenolic extract and its antimicrobial activity. Such is the case of Moreno et al. [89] and Ivanovic et al. [90] who demonstrated that the effectiveness of rosemary is related to a possible synergy between the rosmarinic phenolic acid and the carnosic acid diterpene. Bernardes et al. [91], however, state that there is a close relationship between the concentration levels of the carnosic acid, carnosol diterpenes and the antimicrobial activity of these extracts.

Zaouali et al. [92] reported that, compared with *S. aureus*, antimicrobial activity improves with the presence of α-pinene as a major component. This effect can be correlated with the fact that terpenes can disorganize the cell membrane, and therefore promote the lysis, as stated by Bjapai et al. [93]. The effectiveness of the essential oil of rosemary against *E. coli*, is related to the combined action of the different minority components present in its volatile fraction and should not be associated with the action of any particular component, agreeing with the conclusions published by Zaouali et al. [94]. There are numerous authors who claim that *E. coli, L. monocytogenes* and *S. aureus*, are very resistant bacteria and highlight the importance of chemical composition and proportion between the oil components on their antimicrobial efficacy [94,95].

The antibacterial effect of rosemary has been widely demonstrated in several food studies: beef meatballs [96], cooked beef [97] and in pork sausage [98]. Gomez-Estaca et al. [99] reported that rosemary oil inhibited the growth of common food bacteria contributing to food spoilage. Burt [85] also showed the antibacterial activity of rosemary essential oil against *E. coli*, *Bacillus cereus* and *S. aureus*. In addition, Sirocchi et al. [86] showed that rosemary essential oil inhibited the growth of *Brochothrix thermosphacta* and *Enterobacteriaceae*. Govaris et al. [72] reported an inhibitory effect of dietary supplementation of turkeys with rosemary (5 and 10 g/kg) on the growth of bacteria responsible for spoilage (psychrotrophs, mesophilics, enterobacteria and lactic acid bacteria). Camo et al. [56] also observed an inhibitory effect of the use of rosemary extract added to lamb meat packed in a modified atmosphere in the growth of psychrotrophic bacteria, compared with control meat. This same effect was observed by Quattara et al. [100], however these authors studied pure rosemary essential oil. On the contrary, Ismail et al. [101] showed that rosemary had no antibacterial effect in chicken.

Fernández-López et al. [96], evaluated the antibacterial activity of rosemary extracts in veal meatballs. The results showed a higher antibacterial activity in rosemary extracts, compared to other extracts studied. Only rosemary extracts were able to inhibit the 11 bacteria tested (such as *Lb. lactis FMRD*, *Br. thermosphacta CRA*, *Lb*. *carnosum*, *Br*. *thermosphacta CRA*, *L*. *innocua*, *Lb*. *sake*, *Br*. *thermosphacta CRA*, *Lc*. *mesenteroides* subsp *mesenteroides*, *L*. *monocytogenes*, *Lc*. *mesenteroides* subsp *dextranicum*, *Lb*. *curvatus*).

## 6. Synergistic Effect

It is important to note the possible synergistic effect between rosemary extract and other natural antioxidants. The results shown in the scientific literature offer contradictory conclusions. Resurreccion and Reynolds [102], observed that the joint addition of rosemary extract and tocopherols in meat products failed to increase the antioxidant efficacy of these individually used compounds. On the contrary, Wong et al. [103], concluded that the components of rosemary participate in the regeneration of α-tocopherol, which can be used as substitutes for vitamin C to enhance the stability of vitamin E. Wada and Fang [104], proposed that the synergism between both antioxidants is due to the capacity of the rosemary extract to yield hydrogen atoms to the tocopheryl radicals. This fact justifies the results obtained by Fang and Wada [37], who observed that the antioxidant activity of the α-tocopherol-rosemary mixture on a model fish system was significantly higher than that exhibited by the individually added products. In addition, these authors found that the α-tocopherol molecule remained stable for 10 more days when administered together with rosemary extract.

In this same line, Lai et al. [105] and Stoick et al. [59] showed the existence of a synergistic effect between sodium tripolyphosphate (STTP) and rosemary oleoresin (OR). The combination of OR/STTP was as effective as the application of the BHT-BHA or STPP/TBHQ mixtures during the prevention of WOF (warmed-over flavor) in pre-cooked beef and pork meats respectively [58].

## 7. Conclusions

The health problems derived from lipid oxidation have attracted the attention of consumers and researchers. Numerous diseases, such as aging, cancer, ischemia and atherosclerosis are linked to dietary and biological lipid oxidation products. In this regard, antioxidant compounds present in rosemary extracts and essential oils, delay lipid oxidation in biological systems and food. However, it is essential to consider that the antioxidant and antimicrobial activity of rosemary depend on the fruiting stage, nature of the extracts, mode of extraction, presence of an inhibitor, presence of a synergistic effect with other components, and the concentration of active extract components. If these aspects are taken into account, the application of this natural extract can be complimented in different food systems such as meat, oils and dressing. In view of its application, rosemary extracts could be used in functional foods, pharmaceutical products, plant products and food preservation. Because rosemary is a cheap, available, and a non-toxic herb, these considerations warrant the introduction of rosemary extracts or essential oils, with high phenolic compound contents, into the food industry.

## Figures and Tables

**Table 1:** Antioxidant effectiveness of herbs and spices, evaluated as complete plant materials in different foods.

Spice, Herb	Food	Antioxidant Effectiveness
Marjoram, black pepper, white pepper, sage, rosemary, nutmeg, corianda	Bacon	Rosemary > sage > nutmeg > white pepper > marjoram [51]
Materials of 32 different plants	Bacon	Rosemary > sage > oreganum > nutmeg > thyme [52]
Materials of 32 different plants	Emulsion oil in water	Clove > turmeric > Jamaica pepper > Rosemary [53]
Materials of 15 different plants	Sausages	Sage > Rosemary > paprika > marjoram > Anís [54]
